# The development of aboriginal brain injury coordinator positions: a culturally secure rehabilitation service initiative as part of a clinical trial

**DOI:** 10.1017/S1463423621000396

**Published:** 2021-09-29

**Authors:** Elizabeth Armstrong, Kathy McCoy, Rebecca Clinch, Maureen Merritt, Renee Speedy, Meaghan McAllister, Kym Heine, Natalie Ciccone, Melanie Robinson, Juli Coffin

**Affiliations:** 1 Foundation Chair in Speech Pathology, Edith Cowan University, Perth, Australia; 2 Executive Director, Neurological Council of Western Australia, Perth, Australia; 3 Aboriginal Brain Injury Coordinator, Neurological Council of Western Australia, Perth, Australia; 4 Aboriginal Brain Injury Coordinator, Geraldton Regional Aboriginal Medical Service, Geraldton, Australia; 5 Aboriginal Brain Injury Coordinator, Neurological Council of Western Australia, Perth, Australia; 6 Healing Right Way Project Manager, Edith Cowan University, Perth, Australia; 7 Community Nurse Consultant, Neurological Council of Western Australia, Perth, Australia; 8 Associate Dean Allied Health, Edith Cowan University, Perth, Australia; 9 Aboriginal Research and Engagement Fellow, Murdoch University, Perth, Australia; 10 Ellison Professor Aboriginal Health and Wellbeing, Telethon Kids Institute, the Kimberley, Nedlands, WA, Australia

**Keywords:** aboriginal, aboriginal health worker, care coordination, indigenous, indigenous workforce, integrated care, liaison, rehabilitation, stroke, traumatic brain injury

## Abstract

Brain injury, resulting from stroke and traumatic brain injury, is a common occurrence in Australia, with Aboriginal people affected at a significant rate and impact felt by individuals, families and communities. Access to brain injury rehabilitation services for Aboriginal people is reported to be often limited, with very little support outside the hospital environment. Our research involving Aboriginal brain injury survivors and their families to date has revealed that people often manage ‘on their own’ following such events. Following recommendations from survivors and their families, the Healing Right Way clinical trial, currently underway in Western Australia, has created the role of Aboriginal Brain Injury Coordinator (ABIC) to assist in navigating information and services, particularly after discharge from hospital. Eight positions for this role have been instigated across metropolitan and rural regions in the state. Healing Right Way’s aim is to enhance rehabilitation services and improve quality of life for Aboriginal Australians after brain injury. The ABIC’s role is to provide education, support, liaison and advocacy services to participants and their families over a six-month period, commencing soon after the participant’s stroke or injury has occurred. This paper outlines the development of this role, the partnerships involved, experiences to date and identifies some facilitators and barriers encountered that may impact the role’s ongoing sustainability. Details of components of the planned full Process Evaluation of Healing Right Way related to the ABIC role and the partnerships surrounding it are also provided. In combination with the trial’s ultimate results, this detail will assist in future service planning and provide a model of culturally secure care for stroke and brain injury services that can also inform other sub-acute and primary care models.

## Background

Acquired brain injury, as a result of stroke and traumatic brain injury, affects Australia’s First Peoples (hereafter referred to as Aboriginal people) at a significant rate (Esterman *et al.*, 2018, Katzenellenbogen *et al.*, 2018, Katzenellenbogen *et al.*, 2016, You *et al.*, 2015) and the impact is felt by individuals, families and communities (Armstrong *et al.*, 2015, 2019a, 2019b). However, access to brain injury rehabilitation services for Aboriginal people is reported to be often limited, and there is very little support outside of the hospital environment for the person involved, extended family or community (Armstrong *et al.*, 2015, 2019a, 2019b, Fitts *et al.*, 2019). Our recent research involving Aboriginal brain injury survivors and their families revealed that people often manage ‘on their own’ following such events. Aboriginal brain injury survivors and their families have recommended: i) better access to more (and easily understood) information about brain injury and its consequences, including practical information about supports available after discharge from hospital and ii) community support in navigating rehabilitation services and the recovery ‘journey’ especially during the first six months after the event (Armstrong *et al.*, 2015, 2019a, 2019b). Both brain injury survivors and family members have emphasised the need for and importance of the role of an Aboriginal health professional in providing culturally secure care after brain injury. This is consistent with research outlining the benefits of Aboriginal health professionals to Aboriginal health outcomes in multiple areas such as cardiology, mental health, disability and recommendations for increasing the Aboriginal and Torres Strait Islander workforce (Gilroy *et al.*, 2017, Health Workforce Australia, 2011, Mackean *et al.*, 2020, McKenna *et al.*, 2015). Informed by this previous research, the Healing Right Way project (NHMRC # 1132468), currently underway in Western Australia (WA), is trialling the new role of Aboriginal Brain Injury Coordinator (ABIC) across the state in order to meet this need. The ABIC role was considered a key vehicle for addressing the need for access to information and support in navigating the recovery journey following brain injury. The current paper outlines the development of this position and some preliminary findings to date.

**Figure 1.**
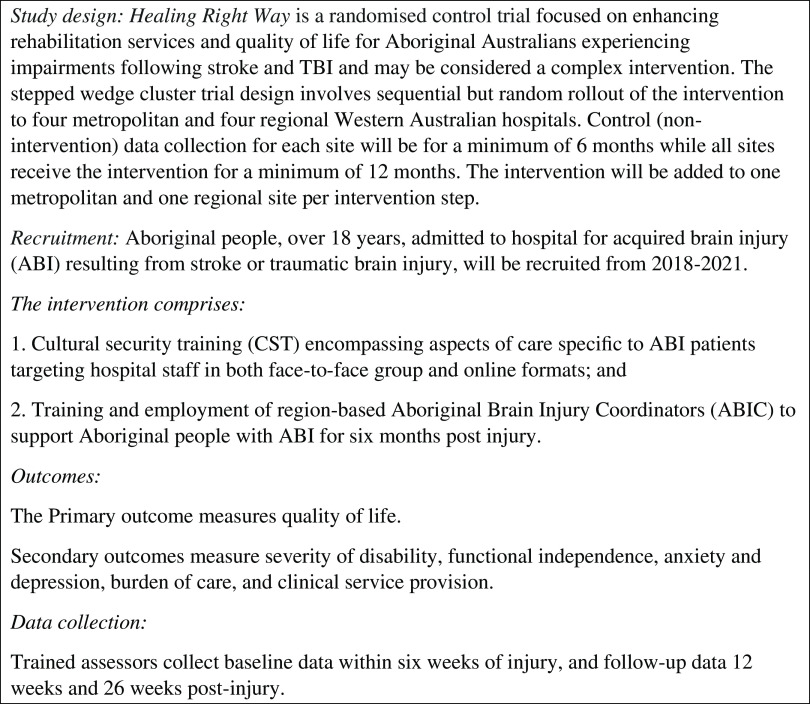
Overview of Healing Right Way.

The ABIC position is a community-based position with the role being to enhance access to rehabilitation services and maximise rehabilitation potential by way of provision of education, support, liaison and advocacy services to Aboriginal brain injury survivors (18 years and over) and their families over a six-month period, commencing within the first six weeks after the participant’s stroke or injury has occurred. The ABIC position is filled by Aboriginal health professionals and is the first position of its kind in the rehabilitation field both nationally and internationally. Healing Right Way is a stepped wedge cluster randomised controlled trial (Brown and Lilford, 2006) operating in eight different sites (four metropolitan and four regional) across WA, with a local ABIC employed at each site (see Figure 1 for overview of the trial). As the role forms part of a clinical trial, the activities of the ABIC role are underpinned and guided by a formal protocol which was written by the research team, in collaboration with project partners outlined below (Armstrong *et al.*, under revision). The trial aims to enhance the cultural security of care provided to Aboriginal people after brain injury, enhance their access to rehabilitation and ultimately improve their quality of life and wellbeing. The study is led by a team of Aboriginal and non-Aboriginal researchers and clinicians. It involves partnerships between the research team, service providers including public hospitals, Aboriginal Community Controlled Health Organisations (ACCHOs), the Neurological Council of WA (a community nursing service) and policy makers including the WA Department of Health and the Stroke Foundation – the national organisation responsible for the development and monitoring of Clinical Guidelines for stroke management in Australia.

**Figure 2.**
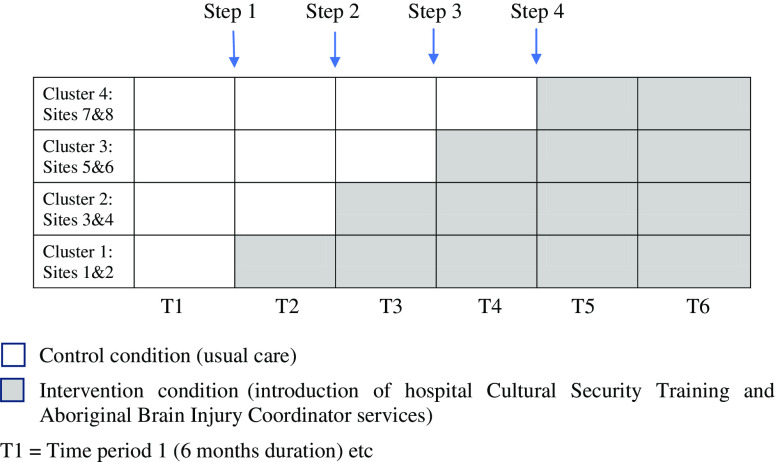
Intervention roll-out across 8 sites as per the stepped wedge cluster design.

The intervention involved in the Healing Right Way clinical trial involves two key components: i) cultural security training for hospital staff at the eight sites participating in the trail and ii) implementation of ABIC positions across the state. Cultural security in the hospital context refers to a state of service delivery in which Aboriginal cultural values and world views are respected, and hospital processes ensure that cultural rights, values and expectations of Aboriginal patients and their families are not compromised (Coffin, 2007). A summary of the training is described in the study protocol (ACTRN # 12618000139279) and in Armstrong *et al.* (under revision 2021). As part of the stepped-wedge cluster design of the trial (see Figure 2), the cultural security training and commencement of the ABIC positions occur concurrently at each site as they reach their intervention phase of the study. Once a site enters the intervention phase of the study, recruited patients are eligible for the ABIC service. This paper aims to describe the development of the ABIC position including implementation of eight such positions across the state, barriers and facilitators experienced to date, formal evaluation mechanisms planned and implications for future services and sustainability.

## Development of the aboriginal brain injury coordinator role

As noted above, the ABIC role grew out of Aboriginal brain injury survivors’ and their families’ recommendation for an Aboriginal health professional to assist in navigating services post hospital discharge. As a partner in the trial, the Neurological Council of Western Australia (NCWA) provided the model of care for the ABIC position, based on its Community Neurological Nurse and Neurocare positions (McCoy and Chan, 2015).

Neurocare is a community-based neurological nursing service available in WA. It draws upon the hub-and-spoke model of The Walton Centre, UK, where community-based neurology nurses with links into hospitals support patients with neurological conditions (Jack *et al*., 2010). This model has shown positive impact on avoidable primary care, ED and outpatient attendances (Riley, 2017). In Neurocare, Integrated Community Neurological Nurses participate in hospital multidisciplinary team discharge planning and support discharge home via care coordination, health navigation, neurological nursing assessment and care in the patient’s home. The service is geared towards shared decision making with patients and patient self-management. In this inward–outward model of care, modes of care delivery include pre-discharge hospital visit, home visit/s, telephone consult/s, other telecommunications (email, text messaging) and telehealth video conferencing (Pugh *et al*., in Press). The point of difference that makes Neurocare contemporary and well aligned with the WA reform agenda (Sustainable Health Review, 2019) is its focus on equity of access and equity of values-based outcomes. Its model is focused on holistic needs of the person rather than a diagnostic label.

Discussions occurred between the academic research team and the NCWA over a period of twelve months in order to explore the Neurocare model and ways in which it could best be implemented across the state within the context of a clinical trial aimed at enhancing access to rehabilitation for Aboriginal people. Discussions also occurred with Aboriginal Community Controlled Health Organisations (ACCHOs) in each of the potential study sites as team support from Aboriginal colleagues for the ABIC positions was clearly essential, with potential for embedding the positions within these services (the ACCHOs) also discussed. NCWA have a longstanding commitment to partnerships, innovation and improving patient outcomes, systems and models. As such the team at NCWA was keen to partner with this project as it was clear that there was much to offer in terms of model development as well as strong governance processes. Recommendations emerging from discussions included that NCWA would recruit new staff to fill the ABIC positions (as there were no Aboriginal staff members), while ACCHOs varied from wanting new/separate staff to fill the positions (due to overload of existing staff) to suggesting that existing members of staff be employed as ABICs, with the role perhaps even spread across 1–2 people to assist capacity building within current staffing. Modelling around the number of participants expected to be enrolled in the study indicated that the ABIC service required a one day/week position for every site involved in the Healing Right Way study.

The governance of the ABIC role is needed to cover operational, clinical and research aspects, and the division of responsibility across project partners (academic research team, NCWA, ACCHOs) was negotiated at every site/region involved in the study. Governance and support structures were critical components of the project, and NCWA was able to provide support, through recruitment, supervision, observation opportunities, skill attainment and use of appropriate processes and assessments, particularly for the ABICs based at their office. The academic research team developed the necessary protocol to guide such things as number and nature of contacts with study participants and the participant and non-participant activities that the role was to encompass. The research team also largely took responsibility for supporting ABICs in research-related processes and in some operational activities, especially while the ABICs were in the orientation and induction phase of their employment. The ACCHOs involved were to provide local operational and general support for the ABICs.

Collegial and clinical support was planned for all ABICs via formal monthly meetings (held via video linkup) which involved the ABICs, an NCWA Community Nurse Consultant and members of the academic research team. Informal supports for clinical queries and those relating to research processes (e.g. data entry) were to be provided by the NCWA and the academic research team regularly and whenever this was requested by the ABIC. The importance of providing cultural support for the ABICs was identified from the outset. This was planned to occur through regular meetings between the ABICs as noted above and through support from local teams at participating ACCHOs.

## Implementation planning

As part of the stepped wedge design of the trial (interventions being introduced at each site on a regular schedule), the ABIC positions were planned to roll out gradually across the state over a two year period, with an ABIC ultimately allocated to a one day/week position at each of the eight sites. Metropolitan and regional sites were paired so that one metropolitan and regional position was filled every six months over the two-year period.

ABICs were to be located within Aboriginal Community Controlled Health Organisations in the regional sites and at NCWA in the metropolitan centre. The ABIC role was advertised on job websites, the website of professional organisations (e.g. the Congress of Aboriginal and Torres Strait Islander Nurses and Midwives), in local newspapers and via circulation of an invitation for expressions of interest via the networks of the ACCHOs and research team.

Guided by the experience of NCWA, the qualifications for applicants were set at a minimum Certificate 3 in a relevant field of health or disability, with preference given to people with qualifications in Aboriginal Health Work, Enrolled or Registered Nursing. Twelve hours of initial training covering the foundations of the role (project background; project team; roles and responsibilities; clinical and research processes; protocol-guided activities; introduction to stroke, brain injury and rehabilitation concepts) formed the basis of the planned training for the ABIC role. A written manual was produced to support this training and is a reference guide for the ABICs to use throughout their employment. The NCWA Community Nurse Consultant would provide additional training input in the area of assessments, community services, identifying neurological needs and support services. This was to be undertaken by way of case studies and case discussion. ABICs were also to be supported to access local education/development opportunities and available online training (e.g. in the area of stroke).

Clinical supervision and support were to be provided as above. A guide for presenting case studies at team meetings was developed by the team to support the ABICs in this regard. Between these sessions, the NCWA Community Nurse Consultant and the academic research team would make themselves available for any clinical or research-related queries raised by the ABICs, via in-person discussion, telephone calls or email as well as group-facilitated discussions.

For ABICs based at NCWA, specific Human Resource and Operations support was to be provided with emphasis on employee safety given there is a sole worker/community worker obligation. For ABICs located in ACCHOs, local operational support would be offered. For metropolitan ABICs employed by NCWA, the ABIC role was to be employed on the same terms and conditions as all NCWA employees. Conditions for rural and remote ABICs were to be based on ACCHO guidance.

### Process evaluation plan

**Figure 3.**
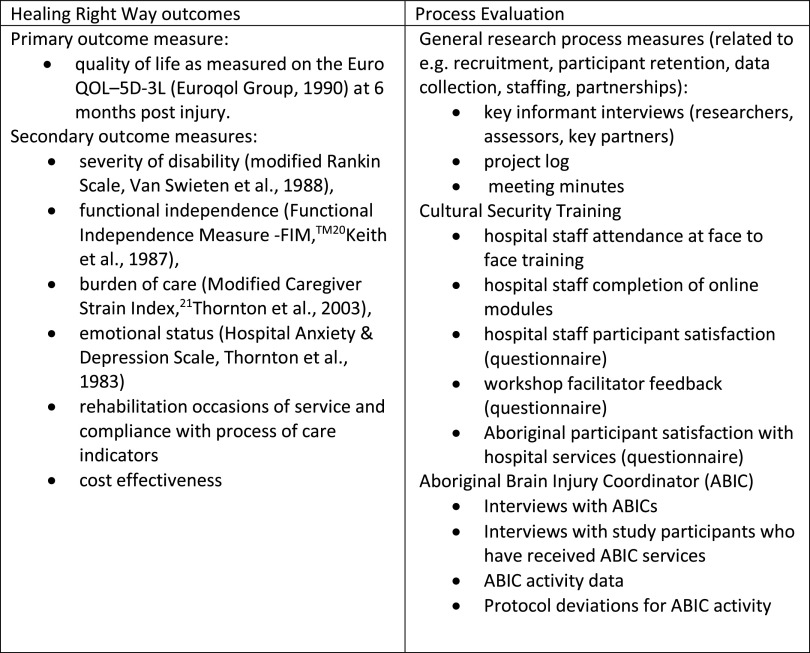
Main trial and process evaluation data.

A formal Process Evaluation (as recommended for clinical trials by the UK Medical Research Council (2006)) was planned to accompany the trial in order to ‘inform and refine the Healing Right Way intervention within the confines of an RCT,’ as well as to ‘provide retrospective analysis to support interpretation of the outcomes of Healing Right Way’ (see the full protocol for the process evaluation in Skoss *et al.* (in press)). This Evaluation will relate to all components of the trial including the cultural security training and the ABIC positions, as well as overall trial processes such as recruitment, participant retention, data collection and maintenance of partnerships (see Figure 3 for overview). Evaluation of processes related to the ABIC positions includes recording details of age, gender, qualifications and prior work experience of each ABIC, time frame in which each ABIC is employed in the study, and staff turnover patterns. Importantly, the ABICs will be invited to be interviewed at the completion of their involvement in the study about their experiences and perspectives on domains including the position itself and activities involved, their workplaces and ways the role could potentially evolve. During the trial, Aboriginal participants receiving the assistance of ABICs are asked to complete questionnaires at 12 and 26 weeks post their injury (facilitated by an Aboriginal research assistant) regarding their experience with ABICs. A subset of participants will also be interviewed. Academic research staff and employers (NCWA and ACCHOs) respond to questionnaires regarding the research collaboration which obviously encompasses the ABIC positions. The formal detailed Evaluation is ongoing and will not be documented in this paper due to the nature of the study being an RCT with final results reserved until the study’s completion. However, as co-authors of this paper, three ABICs, academic researchers (including project Investigators and the project manager) and ABIC employers give their preliminary perspectives regarding their experiences to date in order to provide both an overview of the positions and issues raised in such a novel endeavour. The content below hence does not pre-empt the final Process Evaluation, but rather provides some initial feedback on the structures outlined above in the planning of these positions.

## Ethics

Ethics approval for the Healing Right Way clinical trial including the establishment of the ABIC role was obtained from the Royal Perth Hospital (the lead ethical review committee for the study #125), St John of God Health Care (#1198), Edith Cowan University (#17 291) and the WA Aboriginal Health (#794) Human Research Ethics Committees.

## Findings to date

At the time of writing, 49 participants in the study have received the services of the ABICs since the first ABIC was employed in February 2019 when the first sites entered the intervention phase of the study. This includes participants living in metropolitan, rural and remote areas. The study protocol supports ABIC contact with participants via in person visits, telephone and telehealth, and to date, significant use has been made of these latter modes of contact. Of note, the COVID-19 pandemic has forced suspensions of in-person visits to participants at particular times during the study to date.

### Recruitment of the aboriginal brain injury coordinators

Eight ABIC positions have been created to reflect the eight sites/hospitals involved in Healing Right Way (four metropolitan and four regional sites). The positions are one day/week and aligned with a specific site/hospital. All positions have been filled throughout the study. At the time of the writing of this paper, five ABICs are employed. There are two ABICs based in the metropolitan area (based at NCWA) covering the four sites/hospitals between them (working two days a week each). Another three ABICs work in different regional areas and cover their local site/hospital. Recruitment is currently underway for a regionally based position that became vacant due to a recent resignation.

To date, three positions (one metropolitan and two regional) have been filled, with the ABICs subsequently resigning from the position. Reasons for resignation are being explored via the formal process evaluation which will be reported on at the completion of the trial as noted above. An interim arrangement during such staff vacancy periods has been for another of the ABICs to work with participants from that hospital/site until a local ABIC is recruited, often supporting the participant via telephone contacts. Of the eight ABICs, the study has employed, four have been enrolled nurses, three have had certificates in Aboriginal and/or Torres Strait Islander Primary Health Care Practice and one had three years prior experience working as an Aboriginal Liaison Officer in a rural hospital.

While the research team was initially advised that part time staff would be hard to find, this has in fact not always been the case. Applicants have included people seeking part-time work for family reasons and to accommodate studies they were pursuing at the time. To date, all have been female, with a minimum of 2 years relevant experience in health/community work.

### Establishment of the role

As a new role with a specific focus on brain injury and rehabilitation for Aboriginal people and within the context of a clinical trial, the ABICs, employers and research team all acknowledge the developing nature of this role. One particular challenge has been finding the balance between direct participant contact and non-participant work related to the developing of networks, advocacy and accessing information for participants. Due to a slower than anticipated recruitment rate of participants, initially some ABICs had to focus more on the non-participant activities. This involved attending local hospital/health centre meetings, giving talks to staff about the study and the ABIC role, documenting local and/or state/national resources that may be relevant for people with brain injury. The ease with which these could be embarked upon depended on factors such as the ABIC’s familiarity and contacts within the local community, familiarity with hospitals and rehabilitation related services, and technological access and abilities (access to information from websites, etc). The ABICs reported that it took time to familiarise themselves with the role and responsibilities of both the practical day to day activities as well as the research implications of following a particular protocol, with one ABIC reporting ‘*Hard at the beginning, getting used to everything. Getting used to the logistics. Questioning if I’m doing it right. Learning the protocol.*’ Time was needed to familiarise with the both the role and the research project processes.

### Supervisory/peer support

The ABICs report feeling supported by NCWA and the academic research team during the regular supervision sessions and being part of a team in their workplace. However, one of the most important forms of support comes from the other ABICs. While coordinating days of work to come together is sometimes a challenge, all report the significant value of hearing others’ experiences and getting suggestions from other Aboriginal professionals with similar life experiences and worldviews – having a cultural connection – having lived some of the same experiences, having similar lifestyles and being raised in a similar way – see Table 1. A need for more formal mentoring (cultural and professional) emerged, with the nature of how this support is provided evolving as the study progresses. The previous monthly meetings of the ABICs are now held fortnightly and provide one forum for cultural support between the ABICs themselves. In the metropolitan centre (at NCWA), there are now two ABICS in position, and they are working on the same days each week. Those ABICS have reported that this is of great personal and professional benefit. The ABIC team also keep in contact outside of the scheduled meetings, creating a network of support. An Aboriginal mentor (author 8) was also identified to provide cultural support as needed and participate in the ABIC meeting or conduct a separate group meeting as felt to be appropriate by the ABICs.

**Table 1.** Aboriginal Brain Injury Coordinators’ perceived benefits of group supervision sessions

‘Good to catch up and have a yarn to see how everybody is going. When you yarn things come out and you like ‘you could do this’. Not formal.’ ‘Hearing other people’s ideas on how to do things and you think “I can do that.”’ ‘Encouraging to show you that you can do the role. So when you have doubts about whether you can do it, you go to the meeting and realise you are doing fine, you’re doing ok.’ ‘Hearing where everyone else is at, checking in, being a support system for each other’ ‘Connection with people outside of Perth. Calling and email contact. Having connections with the other ABICs is really good. Coming up with other ideas, that one person might not have come up with, that are culturally appropriate. Like a family to talk and help each other out.’ ‘Connection with people in the same role.’

### The importance of networks

As noted above, the ABICs have supported participants to date in both metropolitan and regional/rural contexts. A significant challenge encountered has been reaching participants when contact attempts via the telephone and addresses available were not successful. Typically hospitals may only have 1–2 contact numbers. Reasons for delayed or unsuccessful contact attempts so far are manifold and so far have included participant availability, changing of participant telephone numbers and access to telephones with available credit and participants moving for care purposes. In these instances, the ABICs have utilised their own community networks and contacted a wide range of service providers to locate and stay connected with participants over the six-month period. The ABICs report that this process has enabled them to build useful networks, which have enabled them to be more able to contact brain injury survivors than is typical for centre-based health services that function within time and activity limitations and are largely staffed by non-Aboriginal health professionals. In some cases, follow-up from other health services has not occurred, and the ABICs have reported participants telling them that they are the only health service they (the participant) have had contact with since discharge from hospital. A small number of participants experiencing homelessness have necessitated particularly creative and extensive networking in order for the support of the ABIC to reach and be of benefit to the participant (see Table 2). The ABICs noted their own surprise at services lacking for people with brain injury: ‘*The role has opened my eyes to the gaps in health services. It’s 2020 and it’s still hard to get services now. People get forgotten about*.’

**Table 2.** Aboriginal Brain Injury Coordinators’ report of homeless participant transferred back from the metropolitan hospital to his rural town

‘He had a lot of issues going on in he’s life before he had the stroke and came on board with the Healing Right Way project, such as moving around a lot, and not having stable accommodation, participant was on the verge of homelessness, participant also had no contact number only of a caseworker that worked at a help centre.
My only way of contact was through the help centre, without any luck of making contact with the participant, due to my work days or simply because the participant was not there at the help centre when I called, I starting making other contacts throughout the health community, the local AMS, the community nurse at the hospital, the social worker and the ALOs, to try and contact the participant. I recall only having one contact with the participant, to which the caseworker from the help centre had contacted me when this particular participant had came into the help centre, whilst talking to one of the contacts that I had made they stated that for some reason the participant had been taken off the follow up appointment register, and that they would be re-registering the participant, so it was nice to know that the calls and emails that I had done would benefit the participant. Just knowing that they are not lost in the system is good.’

At times, during staff vacancy periods, ABICs outside the participant’s residential region have provided the support to that person. Due to the distances involved, this support has been provided by telephone and has required extra work from the ABIC in familiarising herself with local services. While this has been successfully done with several participants to date, a local support is preferable and forms part of the study protocol. The impact of these challenges – challenges which are experienced by many existing health services based regionally or not so – upon participant outcomes and role satisfaction for the ABICs will be taken into consideration when the study’s results are ultimately analysed.

### Mode of interaction

In terms of mode of interaction, the ABICs, and many Aboriginal people, report they prefer a face to face interaction: ‘*Body language is important. Anyone can tell you anything on the phone*’ (ABIC quote). However, phone contact has been noted to be considered useful, and much work has taken place in this way out of necessity. In their work with participants, the ABICs emphasise the importance of a culturally appropriate way to connect and exchange information. This includes the notion of ‘yarning’ (Bessarab and Ng’andu, 2010). In terms of concepts related to yarning in a clinical context – ‘clinical yarning’ (Lin *et al.*, 2016) – ABIC reports of getting to know the holistic context of life for the participant involved could align best with what Lin *et al.* have referred to as ‘social yarning’ (see Table 3), while finding out about impairments and the functional impacts of the brain injury (the ABICs also refer to the ‘Neurological Needs Checklist’ (Government of Western Australia, 2016) could align with ‘diagnostic yarning’ (see Table 3). At times there have been challenges in finding out required information in a culturally safe way. For example, a female health professional asking an Aboriginal man about such things as personal care may be seen, in some communities, as inappropriate, especially if the interaction is between a senior or Elder Aboriginal man and a young Aboriginal woman.

**Table 3.** Aboriginal Brain Injury Coordinators’ comments on social and diagnostic yarning

‘You just ring up about- how you going - then it turns into a yarn then they start talking and you get to know them.’ ‘Once you introducing yourself and start talking, where the participants start to feel comfortable and you start to feel comfortable, that’s where you start to found out thing family history it’s a good starting point to build a rapport just yarning about thing, you’ll be surprised sometimes what comes out of social yarning.’ ‘You find out a lot through yarning and not always directly going through checklist – however relevant information emerges naturally.’
‘Being female I’m also mindful of the cultural side of thing as well, I always ask if it’s okay and if participants don’t mind me asking them questions. Some questions are a bit personal, so I try to ask in a way that doesn’t make the participants uneasy. Especially to our male participants. That’s where social and diagnostic yarning is important.’

### Interpersonal relationships

As well as their work in service coordination, education and advocacy (as described above), the ABICs describe the importance of interpersonal relationships in the support they provide to participants. Based on verbal feedback/reactions from some participants to date, they feel that the participants want to know that ‘someone cares’ and that there is some form of continuity support offered:

*‘It’s somebody out of their little circle and maybe they’re thinking that there’s someone there that will care about them, support them, they’re not alone, they’re just not limited, someone’s interested in them, concerned about their welfare and health’* (ABIC report)

While in a conversation preparing for this paper, one ABIC queried the difference she was making with a particular participant, as he appeared to be managing relatively well. Her colleague spoke from her own experience:

*‘Oh you’d be surprised what a phone call to someone could mean, somebody to talk to and say hey, how you going, how’s things, means someone cares’*

To which her colleague responded:

*‘And that’s true eh cos once you start talking he doesn’t know when to stop’*

Another ABIC reported on how accepting a participant was of the support she provided, saying:

*‘I said the six months is up and she goes “ will you be calling us again?’ and I was oh ‘the project’s up but I might give you a call see how you’re goin”’ (laughter)*

The ABICs also expressed concern about continuity of services beyond the six months of the clinical trial, worrying ‘have I given them enough to help them in their recovery?’ hoping that other services will be activated and working and hoping to empower people to be in control of their health.

### Health service research collaboration

The ABICs have been embedded in existing teams with varying degrees of success to date. While team support is generally present across the sites, working on a particular research project with a discrete group of ‘clients’ can sometimes isolate the ABIC and their service from the core business of the host agency. For NCWA whose core business ***is*** working with people with neurological conditions, this is less the case. For Aboriginal Community Controlled Health Organisations whose core business is not rehabilitation or brain injury specifically, a degree of isolation (for the ABICs) may be expected. However, as ACCHOs increasingly become service providers for Australia’s new National Disability Insurance Scheme (see May *et al.* (2018) for details), a closer link between the ABIC role and other ACCHOs health workers may evolve and discussions about this are currently underway. As one of the aims of the Healing Right Way study is to make the ABIC positions sustainable beyond the time frame of the research project, their successful embedding within partner organisations is highly desirable.

Shared governance (between the academic research team and health service providers) of the ABIC positions has required ongoing communication in order to ensure alignment of the goals of the research project and service provider expectations. This communication has aimed to ensure all parties are satisfied with the performance of the ABIC role, and the ABICs themselves are clear on lines of communication and line management responsibilities.

## Discussion

The unique role of the ABIC is creating a new model of culturally secure care for Aboriginal people following a brain injury, with some positive feedback from participants to date. The roll-out of the positions across WA suggests that the role is feasible, although ongoing challenges with embedding the positions in existing services continue to be navigated, with ultimate translation and sustainability in mind. The ultimate impact of the ABIC service upon health and well-being outcomes of the participants with brain injury (as measured by the outcome tools used in the Healing Right Way clinical trial) will be determined as part of the study’s findings at the completion of the project. However, the exercise of addressing culturally secure care for Aboriginal brain injury survivors, who currently typically do not receive ongoing rehabilitation services, is providing a unique opportunity to increase the profile of Aboriginal brain injury survivors and explore alternative ways of facilitating access to a variety of services.

Utilising the innovative Neurocare model of ‘in-reach’ to hospitals, followed up by comprehensive community support, provides a practical, client-centred approach to support for Aboriginal people with brain injury. In-person and telehealth modalities for service delivery are being used in the study and will be analysed for impact. Employing Aboriginal health professionals to fulfil these roles to support brain injury survivors acknowledges the necessity of cultural affinities and synergies in being able to supply culturally secure support and is consistent with what Aboriginal brain injury survivors have recommended (Armstrong *et al.*, 2019). The ongoing evolution of the role should assist in the refining of the role capabilities and boundaries, and this will be well captured in the study’s formal Process Evaluation. While the importance of Aboriginal health professionals in improving outcomes for Aboriginal people has been repeatedly emphasised for a number of years (Cheng, 2007, Health Workforce Australia, 2011, Taylor *et al.*, 2009), the Aboriginal health workforce numbers are limited, although recent interest in addressing barriers and facilitators is promising (e.g. Taylor *et al.*, 2009, Wilson *et al.*, 2020). Embedding the role in both Aboriginal and non-Aboriginal organisations in this study provides an opportunity for expanding care options in both types of organisations from different perspectives. For the ACCHOs, it provides opportunity for expanding and developing the skills and knowledge within the organisation for addressing the needs of brain injury survivors (some of whom may be existing clients because of co-occurring conditions). For NCWA, it provides opportunity to further expand its service reach by developing a service that is culturally accessible to Aboriginal clients. In attempting to address previous issues identified as important for implementation of specialised Aboriginal health professional roles (e.g. Taylor *et al.*, 2009) (including training, organisational and cultural support, appropriate financial remuneration and planning for sustainability of these roles in relevant workplaces), Healing Right Way provides a potential model for similar ongoing positions related to multiple conditions and health contexts.

Of significance, but perhaps not surprising, is the emerging emphasis on the utilisation of Aboriginal community networks for following up/providing care for people with brain injury following their discharge from hospital. This is notoriously difficult for non-Aboriginal health professionals with little contact with Aboriginal communities and contributes to ongoing lack of engagement with their Aboriginal patients. It is reflective of the accessibility of the service to Aboriginal people, and Aboriginal Community Controlled Health Organisations are inherently more experienced in this regard. Aboriginal health professionals remain few, especially so in the brain injury sector, and this issue remains unresolved. The implications of poor follow-up and patients discharging themselves from hospitals against medical advice (DAMA) after an acute event such as brain injury and re-presenting at a hospital with further complications, another stroke, etc., are well documented (Katzenellenbogen *et al.*, 2013, Mackean *et al.*, 2020).

The importance of interpersonal communication styles underpinning the care ABICs provide to people with brain injury is also a significant, if unsurprising, finding to date. The success of the naturally employed yarning approach used by the ABICs to develop a trusting relationship and successful exchange of information with participants reinforce suggestions that the frequently used medical discourse is often unsuccessful and indeed contributes to negative outcomes for Aboriginal people interacting with health services. Miscommunication has been identified as a major contributor to ongoing lack of engagement between Aboriginal brain injury survivors and health service providers (Armstrong *et al.*, 2015, 2019b). The simple narrative that ‘Aboriginal patients’ often don’t want or prioritise health services after stroke or traumatic brain injury was de-bunked in previous research (Armstrong *et al.*, 2015, 2019b) and is again not borne out in this study to date.

This paper has aimed to provide details of a new initiative to support Aboriginal people who are recovering from brain injury and their families. While final results of the Healing Right Way trial including the impact of the ABIC position can only be reported at the trial’s conclusion, this paper has attempted to document some emerging issues and experiences that may be of benefit to those planning similar service delivery models. The trial’s formal Process Evaluation component of the study will document the ongoing evolution of the ABIC role and barriers and facilitators involved in its implementation and sustainability. Changing contexts throughout the trial will provide many further insights and also see potential for alignment of the ABIC role with other initiatives such as the National Disability Insurance Scheme (Olney & Dickinson, 2019). The ABIC role may also serve as a model of support roles for Aboriginal people with other conditions. Experience gained from its implementation will be invaluable in addressing other potential initiatives.

## References

[ref1] Armstrong E, Coffin J, Hersh D, Katzenellebogen J, Thompson S, Ciccone N, Flicker L, Woods D, Hayward C, Dowell C and Mcallister M (2019a, online) “You felt like a prisoner in your own self, trapped”: the experiences of Aboriginal Australians with acquired communication disorders. Disability and Rehabilitation 1–14. 10.1080/09638288.2019.1686073. 31692386

[ref2] Armstrong E, Coffin J, Hersh D, Katzenellenbogen J, Thompson S, Flicker L, Mcallister M, Cadilhac D, Godecke E, Rai T, Hayward G, Drew N, Lin I, Woods D and Ciccone N (under revision) Healing Right Way: study protocol for a randomised control trial to enhance rehabilitation services and improve quality of life in Aboriginal Australians after brain injury. BMJ-Open 10.1136/bmjopen-2020-045898 PMC8479943 34588230

[ref3] Armstrong E, Coffin J, Mcallister M, Hersh D, Katzenellenbogen JM, Thompson SC, Ciccone N, Flicker L, Cross N, Arabi L, Woods D and Hayward C (2019b) ‘I’ve got to row the boat on my own, more or less’: Aboriginal Australian experiences of traumatic brain injury. Brain Impairment 20, 120–136.

[ref4] Armstrong E, Hersh D, Hayward C and Fraser J (2015) Communication disorders after stroke in Aboriginal Australians. Disability and Rehabilitation 37, 1462–1469. 25365701 10.3109/09638288.2014.972581

[ref5] Bessarab D and Ng’andu B (2010) Yarning about yarning as a legitimate method in Indigenous research. International Journal of Critical Indigenous Studies 3, 37–50.

[ref6] Brown CA and Lilford RJ (2006) The stepped wedge trial design: a systematic review. BMC Medical Research Methodology 6, 54. 17092344 10.1186/1471-2288-6-54 PMC1636652

[ref7] Cheng MH (2007) Aboriginal workers key to indigenous health in Australia. The Lancet 370, 1533–1536. 10.1016/S0140-6736(07)61648-1 17992724

[ref8] Coffin J (2007) Rising to the challenge in Aboriginal health by creating cultural security. Aboriginal & Islander Health Worker Journal 31, 22–24.

[ref9] Esterman A, Thompson F, Fitts M, Gilroy J, Fleming J, Maruff P, Clough A and Bohanna I (2018) Incidence of emergency department presentations for traumatic brain injury in Indigenous and non-Indigenous residents aged 15–64 over the 9-year period 2007–2015 in North Queensland, Australia. Injury Epidemiology 5, 40. 30417259 10.1186/s40621-018-0172-9 PMC6230543

[ref11] Fitts MS, Bird K, Gilroy J, Fleming J, Clough AR, Esterman A, Maruff P, Fatima Y and Bohanna I (2019) A qualitative study on the transition support needs of Indigenous Australians following traumatic brain injury. Brain Impairment 20, 137–159.

[ref12] Gilroy J, Dew A, Lincoln M and Hines M (2017) Need for an Australian Indigenous disability workforce strategy: review of the literature. Disability and Rehabilitation 39, 1664–1673. 27384020 10.1080/09638288.2016.1201151

[ref13] Government of Western Australia, Department of Health (2016) Neurological Needs Checklist Version 7.0 HPV102062016. Adpated from Philp, I., Brainin, M., Walker, M., Ward, A., Gillard, P., Shileds, A., and Norrving, B. 2012: Development of a Post Stroke Checklist to Standardize Follow-Up Care for Stroke Survivors. Journal of Stroke and Cerebrovascular Diseases, Dec 21, on the internet (cited 24/04/2013). Available from http://www.sciencedirect.com/science/article/pii/S1052305712003606 10.1016/j.jstrokecerebrovasdis.2012.10.016 23265778

[ref14] Health Workforce Australia (2011) Growing our future: final report of the Aboriginal and Torres Strait Islander Health Worker project. Adelaide. http://hdl.voced.edu.au/10707/221503

[ref312] Jack B, Kirton J, O’Brien M and Roe B (2010). Bridging the gap: the impact of a generic neurology nursing service on patients and carers. Poster session presented at Royal College of Nursing (RCN) International Nursing Research Conference, Gateshead, United Kingdom.

[ref17] Katzenellenbogen JM, Atkins E, Thompson SC, Hersh D, Coffin J, Flicker L, Hayward C, Ciccone N, Woods D, Greenland ME, Mcallister M and Armstrong E (2018) Missing voices: profile, extent, and 12-month outcomes of nonfatal traumatic brain injury in Aboriginal and non-Aboriginal adults in Western Australia using linked administrative records. Journal of Head Trauma and Rehabilitation 33, 412–423. 10.1097/HTR.0000000000000371 29601340

[ref16] Katzenellenbogen JM, Atkins ER, Thompson SC, Hersh D, Coffin J, Flicker L, Hayward C, Ciccone N, Woods D, Mcallister M and Armstrong E (2016) Missing voices: profile and extent of acquired communication disorders in Aboriginal and non-Aboriginal adult stroke survivors in Western Australia using linked administrative records. International Journal of Stroke 11, 103–116. 26763026 10.1177/1747493015607521

[ref18] Katzenellenbogen JM, Sanfilippo FM, Hobbs MS, Knuiman MW, Bessarab D, Durey A and Thompson SC (2013) Voting with their feet--predictors of discharge against medical advice in Aboriginal and non-Aboriginal ischaemic heart disease inpatients in Western Australia: an analytic study using data linkage. BMC Health Services Research 13, 330. 23962275 10.1186/1472-6963-13-330 PMC3765140

[ref19] Keith RA, Granger CV, Hamilton BB and Sherwin S (1987) The functional independence measure: a new tool for rehabilitation. Advances in Clinical Rehabilitation 1, 6–18. 3503663

[ref20] Lin I, Green C and Bessarab D (2016) ‘Yarn with me’: applying clinical yarning to improve clinician–patient communication in Aboriginal health care. Australian Journal of Primary Health 22, 377. 28442021 10.1071/PY16051

[ref21] Mackean T, Withall E, Dwyer J and Wilson A (2020) Role of Aboriginal Health Workers and Liaison Officers in quality care in the Australian acute care setting: a systematic review. Australian Health Review 44, 427–433. 31931950 10.1071/AH19101

[ref22] May T, Roberts J, Webber M, Spreckley M, Scheinberg A, Forrester M and Williams K (2018) Brief history and user’s guide to the Australian National Disability Insurance Scheme. Journal of Paediatrics and Child Health 54, 115–120. 29030948 10.1111/jpc.13748

[ref23] Mccoy K and Chan H (2015) A neurological integrated care pathway. Australasian Journal of Neuroscience 26, 1–6.

[ref24] Mckenna B, Fernbacher S, Furness T and Hannon M (2015) “Cultural brokerage” and beyond: piloting the role of an urban Aboriginal Mental Health Liaison Officer. BMC Public Health 15, 881. 26358718 10.1186/s12889-015-2221-4 PMC4566419

[ref25] Olney S and Dickinson H (2019) Australia’s new national disability insurance scheme: implications for policy and practice. Policy Design and Practice 2, 275–290. doi: 10.1080/25741292.2019.1586083.

[ref313] Pugh, JD, McCoy K, Needham M, Jiang L, Giles M, McKinnon E and Heine K (in Press). Evaluation of an Australian neurological nurse-led model of postdischarge care. Health and Social Care in the Community. 10.1111/hsc.13498 34245179

[ref314] Riley J (2017) The neuro network program. Advances in Clinical Neuroscience & Rehabilitation (ACNR) 16, 19.

[ref315] Skoss, R., White, J., Stanley, M., Robinson, M., Thompson, S., Armstrong, E. and Katzenellenbogen, J. (in press): Study protocol for a prospective process evaluation of a culturally secure rehabilitation program for Aboriginal Australians after brain injury: the Healing Right Way project. BMJ-Open. 10.1136/bmjopen-2020-046042 PMC8479980 34588232

[ref28] Sustainable Health Review (2019) Sustainable health review: final report to the Western Australian government. Western Australia: Department of Health.

[ref29] Taylor KP, Thompson SC, Smith JS, Dimer L, Ali M and Wood MM (2009) Exploring the impact of an Aboriginal Health Worker on hospitalised Aboriginal experiences: lessons from cardiology. Australian Health Review: A Publication Of The Australian Hospital Association 33, 549–557. 20166903 10.1071/ah090549

[ref30] The EuroQol Group (1990) EuroQol-a new facility for the measurement of health-related quality of life. Health Policy 16, 199–208. 10109801 10.1016/0168-8510(90)90421-9

[ref311] Thornton M and Travis SS (2003) Analysis of the reliability of the modified caregiver strain index. The Journals of Gerontology Series B: Psychological Sciences and Social Sciences 58, S127–S132. 10.1093/geronb/58.2.s127 12646602

[ref31] UK Medical Research Council (2006) Developing and evaluating complex interventions. www.mrc.ac.uk/complexinterventionsguidance

[ref32] Van Swieten J, Koudstaal P, Visser M, et al. (1988) Interobserver agreement for the assessment of handicap in stroke patients. Stroke 19, 604–607. 3363593 10.1161/01.str.19.5.604

[ref33] Wilson AM, Kelly J, Jones M, O’donnell K, Wilson S, Tonkin E and Magarey A (2020) Working together in Aboriginal health: a framework to guide health professional practice. BMC Health Services Research 20, 601. 32611413 10.1186/s12913-020-05462-5 PMC7329497

[ref34] You J, Condon JR, Zhao Y and Guthridge SL (2015) Stroke incidence and case-fatality among Indigenous and non-Indigenous populations in the Northern Territory of Australia, 1999–2011. International Journal of Stroke 10, 716–722. 25588511 10.1111/ijs.12429

[ref35] Zigmond AS and Snaith RP (1983) The hospital anxiety and depression scale. Acta Psychiatrica Scandinavica 67, 361–370. 6880820 10.1111/j.1600-0447.1983.tb09716.x

